# Clinical characteristics of respiratory tract infection caused by *Klebsiella pneumoniae* in immunocompromised patients: a retrospective cohort study

**DOI:** 10.3389/fcimb.2023.1137664

**Published:** 2023-08-16

**Authors:** Yahui Liu, Lin Huang, Jing Cai, Haixing Zhu, Junjie Li, Youchao Yu, Yumin Xu, Guochao Shi, Yun Feng

**Affiliations:** ^1^ Department of Respiratory and Critical Care Medicine, Ruijin Hospital Affiliated Shanghai Jiao Tong University School of Medicine, Shanghai, China; ^2^ Institute of Respiratory Diseases, School of Medicine, Shanghai Jiaotong University, Shanghai, China; ^3^ Department of Pulmonary and Critical Care Medicine, Haining People’s Hospital, Jiaxing, China; ^4^ Department of Hospital Infection Management, Department of Infectious Diseases, Ruijin Hospital, School of Medicine, Shanghai Jiao Tong University, Shanghai, China

**Keywords:** *Klebsiella pneumoniae*, immunocompromised patients, respiratory tract infection, sputum culture, case fatality rate

## Abstract

**Purpose:**

With advancements in medical technology and the growth of an aging society, the number of immunocompromised patients has increased progressively. *Klebsiella pneumoniae* (*K. pneumoniae*) is one of the most common opportunistic pathogens, causing a severe disease burden. We aimed to further clarify the differences in respiratory tract *K. pneumoniae* infections between immunocompromised and immunocompetent populations.

**Methods:**

We retrospectively compared cases of respiratory tract *K. pneumoniae* infection in immunocompromised and immunocompetent patients admitted to Ruijin Hospital in Shanghai between January 2019 and August 2020 to clarify the differences between the two groups.

**Results:**

We enrolled 400 immunocompromised patients and 386 immunocompetent patients. Compared to the immunocompetent group, immunocompromised patients were more likely to develop bacteremia and shock and to require mechanical ventilation support during hospitalization. Immunocompromised patients also had a greater probability of polymicrobial infection and a higher rate of antibacterial resistance to carbapenem, which resulted in a higher intensive care unit admission rate, 30-day case fatality rate (CFR), and 6-month CFR. Multivariate analysis indicated that immunocompromised patients with respiratory diseases (odds ratio [OR], 2.189; 95% confidence interval [CI], 1.103-4.344; *P* = 0.025) and cardiovascular diseases (OR, 2.008; 95% CI, 1.055-3.822; *P* = 0.034), using mechanical ventilation (OR, 3.982; 95% CI, 2.053-7.722; *P* = 0.000), or infected with multidrug-resistant *K. pneumoniae* (OR, 3.870; 95%, 1.577-9.498; *P* = 0.003) were more likely to have a higher 30-day CFR.

**Conclusion:**

The disease burden of *K. pneumoniae* infection in immunocompromised patients is high. Immunocompromised patients who presented with respiratory diseases and cardiovascular diseases, used mechanical ventilation, or were infected with multidrug-resistant *K. pneumoniae* experienced a higher 30-day mortality rate.

## 1 Introduction

*Klebsiella pneumonia* (*K. pneumoniae*) is one of the most commonly found Gram-negative bacilli in hospital environments, and is primarily considered an opportunistic pathogen that threatens vulnerable populations. However, with the advancement of life sciences and medical care, the average life expectancy has increased, and the number of immunosuppressed people has gradually grown, allowing infections caused by *K. pneumoniae* in the community to occur occasionally (Lin et al., 2010; Melot et al., 2015; Juan et al., 2019). Additionally, compared to other Gram-negative bacilli, reported resistance proportions to third-generation cephalosporins were higher in cases of *K. pneumoniae* infection (Antimicrobial resistance: global report on surveillance). Hypervirulent multidrug-resistant (MDR) strains of *K. pneumoniae* have also gradually emerged and spread widely (Zhang et al., 2016; Lee et al., 2017; Shankar et al., 2018). Infection by *K. pneumoniae* can cause sepsis, urinary infection, and pneumonia, resulting in a heavy disease burden on society (Zhang et al., 2016; Lee et al., 2017; Shankar et al., 2018). It was reported that the mortality rate for *K. pneumoniae* hospital-acquired pneumonia can exceed 50% sometimes (Antimicrobial resistance: global report on surveillance).

Owing to the unremitting efforts of clinicians and others, guidelines about clinical aspects of infectious diseases are well documented and continuously improved. However, due to the nature of the disease itself or the use of various immunosuppressive treatments, the causative pathogens, clinical characteristics, treatments, and infection prognosis in the immunocompromised population differ significantly from those of the immunocompetent population (Di Pasquale et al., 2019). Many of the existing guidelines are mostly based on immunocompetent populations and may not be applicable to immunocompromised populations (Ramirez et al., 2020). Therefore, we believe that it is of great significance to discover the characteristics of infection in immunocompromised people, especially the aspects that are different from those with normal immune function, and summarize the key points of treatment.

Considering the burden of *K. pneumoniae* infection in the immunocompromised population, it is crucial to obtain accurate and recent data concerning both the clinical and microbiological characteristics of *K. pneumoniae* infection in the immunocompromised population, as well as the predictors of intensive care unit (ICU) admission and the case-fatality rate (CFR). Our primary study objective was to identify the differences in respiratory tract infections by *K. pneumoniae* between immunocompetent and immunocompromised individuals. The secondary objective was to determine the risk factors of respiratory tract infection by *K. pneumoniae* in immunocompetent and immunocompromised populations so as to achieve early detection, early prevention, and an improved prognosis.

## 2 Methods

### 2.1 Study design and population

This retrospective cohort study was conducted at Ruijin Hospital, Shanghai Jiaotong University School of Medicine, China. Microbiology records were used to identify patients with respiratory tract infection caused by *K. pneumoniae.* The medical records of patients with respiratory tract infections involving *K. pneumoniae* were reviewed. A respiratory tract infection was defined by the following measures: (1) an acute pulmonary infiltrate evident on a chest X-ray or computerized tomography scan and compatible with pneumonia and (2) confirmatory findings of the clinical examination (Lin et al., 2010). Respiratory tract infection by *K. pneumoniae* was defined by the presence of as least one sputum culture sampled yielding a pathogen presumed to be the cause of the infection. The diagnosis was reconfirmed by two infectious disease specialists. For patients with multiple episodes of *K. pneumoniae* infection during the study period, only the first episode was included.

### 2.2 Data collection

Clinical information of enrolled patients including demographic characteristics, comorbidities, immune function, surgery or invasive operation, biochemical indicators, microbiological characteristics, treatments, and outcome, were collected. Sputum samples were collected for culture, and pathogens were identified using standard microbiological procedures. Homogenized sputum samples were spread onto plates containing blood agar/chocolate agar (non-selective growth), Columbia CNA agar (selective Gram-positive), MacConkey agar (selective Gram-negative), or Brucella agar (for culture of anaerobes) and incubated at 35°C in the appropriate atmosphere. Susceptibility testing was performed by standard agar dilution methods. Results were interpreted according to the Clinical and Laboratory Stands Institute susceptibility breakpoints (CLSI, 2021). Bacterial colonies proceeded to the semi-quantitative steps. In order to report bacterial load, culture plates were divided into four quadrants that were then systematically streaked from the first to the last quadrant using an inoculation loop. After incubation, the number of quadrants with bacterial growth was assessed. A result of “negative” was recorded if no growth was observed on the plate, while “1+” was reported if growth was only observed in the first quadrant; “2+” was reported if growth was observed in the first and second quadrants; “3+” was reported if growth was observed in the first, second, and third quadrants; and “4+” was reported if growth was observed in all four quadrants of the plate, respectively. Improvement of *K. pneumoniae* infection was defined by a sputum culture turning negative from positive or positive sputum cultures being reduced by two plus signs “+”. Outcome variables included ICU admission, 30-day CFR, 6-month CFR and length of stay (LOS) (Lin et al., 2010; Juan et al., 2019; Montrucchio et al., 2022).

For this non-interventional study, a waiver for medical ethical approval was granted by the Ethics Committee of Ruijin Hospital, Shanghai Jiao Tong University School of Medicine. The study was performed in accordance with the ethical standards as laid out in the 1964 Declaration of Helsinki. No patient consent was required due to the retrospective nature of the study.

### 2.3 Definition of immunocompromised patients

Participants were classified into the immunocompetent and immunocompromised groups. Patients were defined as immunocompromised when as least one of the following conditions were present: (1) asplenia; (2) active malignancy, or receiving cancer chemotherapy or radiotherapy during the last 3 months; (3) HIV infection with a CD4+ lymphocyte count < 200 cells/μL or percentage < 14%; (4) solid organ transplantation or hematopoietic stem cell transplantation; (5) receiving corticosteroid therapy with a ≥ 20 mg dose of prednisone or equivalent daily for ≥ 14 days or a > 700 mg cumulative dose of prednisone; (6) receiving biologic modulators; (7) receiving disease-modifying anti-rheumatic drugs or other immunosuppressive drugs; (8) liver cirrhosis; (9) severe burns; (10) primary immune deficiency diseases or acquired immune deficiency disorder; (11) hematological diseases, including aplastic anemia, lymphoma, acute or chronic leukemia, or multiple myeloma; and (12) neutropenia, defined by a neutrophil count < 500 cells/μL at complete blood cell count (Di Pasquale et al., 2019; Ramirez et al., 2020; Lin et al., 2022). The immunocompromised state had to be active at the time of the patient’s study inclusion. A neoplastic disease was defined as active if it required medical or surgical intervention within the last year or if no-treatable metastases were present at the time of study enrollment.

### 2.4 Statistical analysis

Outcome variables were compared between immunocompetent and immunocompromised patients. Propensity score matching was used to balance the baseline characteristics of immunocompetent and immunocompromised patients. Categorical variables are described as frequencies (percentages), while continuous variables are presented as mean and standard error of the mean (SEM) values or as median and interquartile range (IQR) values for data not normally distributed (Kolmogorov-Smirnov test). Categorical variables were analyzed using the chi-square test or Fisher’s exact test, as appropriate. Continuous variables were analyzed by *t* test or by non-parametric Mann-Whitney *U* test after verifying a non-normal distribution.

Univariate and multivariate logistic regression analyses were performed to identify variables predictive of disease severity in patients with *K. pneumoniae* infection. The following variables were analyzed: age, sex, presence of other bacterial infections, procalcitonin, C-reactive protein, neutrophil count, creatinine (Cr), alanine aminotransferase, aspartate aminotransferase, gamma-glutamyl transpeptidase (γGT), and respiratory support. Variables with *P* < 0.1 in the univariate analysis, after checking for collinearity, were selected via backward elimination for a multivariate logistic regression model. The fitness of the model was tested using the Hosmer-Lemeshow goodness-of-fit test. All statistical tests were two-tailed and *P <*0.05 was considered to indicate statistical significance. The analysis was completed using the SPSS version 25.0.0.2 statistical package (IBM Corporation, Armonk, NY, USA).

## 3 Results

### 3.1 Baseline characteristics

**Figure 1:**
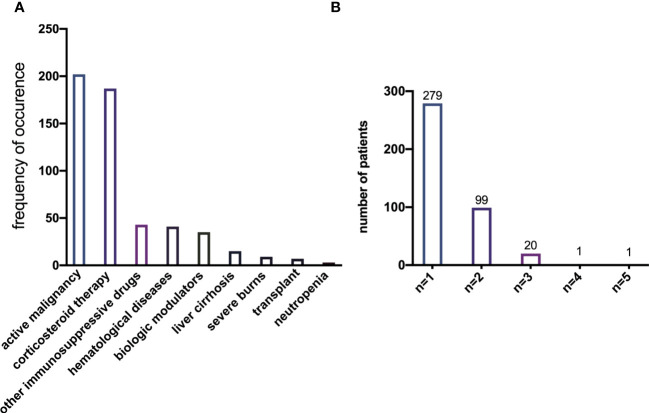
**(A)** Prevalence of each single risk factor for immunocompromise of *K. pneumoniae* infection. **(B)** Prevalence of the number of risk factors present simultaneously in a single patient.

During the study period, data from 786 patients with respiratory infections by *K. pneumoniae* were collected. According to the definition of being immunocompromised, 386 (49.11%) of all enrolled patients were immunocompetent and 400 (50.89%) were immunocompromised. The prevalence of each risk factor for being immunocompromised is depicted in **Figure 1A**, with active malignancy (50.50%) and chronic steroid use (46.75%) being the most frequent risk factors. A total of 121 patients had more than one risk factor for being immunocompromised (**Figure 1B**).

Baseline characteristics of immunocompetent versus immunocompromised patients are shown in **Table 1** and **Supplementary Figure S1**. There were no differences in sex (*P* = 0.7660) and age (*P* = 0.2048) between the groups. Forty patients in the immunocompromised group developed bacteremia during hospitalization. Compared to the immunocompetent group, more immunocompromised patients developed shock (*P* < 0.0001) and received mechanical ventilation support (*P* = 0.0183). In contrast, more immunocompetent patients underwent invasive operations or surgery (*P* < 0.0001). Additionally, more patients in the immunocompromised group had comorbid diseases, such as respiratory diseases (*P* = 0.0043), diabetes (*P* = 0.0265), malignancy (*P* < 0.0001), chronic kidney diseases (*P* = 0.0045), or chronic hepatic diseases (*P* < 0.0001).

**Table 1:** Clinical characteristics of the two study groups (immunocompetent vs immunocompromised).

Characteristic		No./Total (%)		*P* Value
		Immunocompetent patients (n = 386)	Immunocompromised patients (n = 400)	
*Sex*	Male (%)	283 (73.32%)	297 (74.25%)	0.7660
*Age, y*	Mean (SEM)	64.84 (0.7287)	66.19 (0.7738)	0.2048
	<65 y	171 (44.30%)	170 (42.50%)	0.6106
	≥65 y	215 (55.70%)	230 (57.50%)	
*Bacteremia*		0 (0%)	48 (12.00%)	<0.0001
*shock*		3 (0.777%)	43 (10.75%)	<0.0001
*Mechanical ventilation*		105 (27.20%)	140 (35.00%)	0.0183
*Invasive operation or surgery*		259 (67.10%)	163 (40.75%)	<0.0001
*Comorbid conditions*	Nervous system diseases	90 (23.32%)	79 (19.75%)	0.2238
	Respiratory diseases	173 (44.82%)	220 (55.00%)	0.0043
	Cardiovascular diseases	195 (50.52%)	227 (56.75%)	0.0798
	Diabetes	73 (18.91%)	102 (25.50%)	0.0265
	History of solid organ tumor	103 (26.68%)	193 (48.25%)	<0.0001
	Digestive diseases	60 (15.54%)	72 (18.00%)	0.3571
	Chronic kidney disease	21 (5.44%)	54 (13.50%)	0.0045
	Chronic hepatic diseases	10 (2.59%)	41 (10.25%)	<0.0001

SEM, standard error of the mean.

Data represent no. (%) unless otherwise specified.

### 3.2 Microbiological characteristics

Microbiological testing was performed in all the enrolled patients, and the microbiological findings are provided in **Table 2**. Immunocompromised patients were more likely to have polymicrobial respiratory infections, including *K. pneumoniae* (*P* < 0.0001) (**Table 2**; **Supplemental Figure S2**). As is known, *K. pneumoniae* is the main cause of infections initiated by carbapenem-resistant bacteria worldwide (Antimicrobial resistance: global report on surveillance). According to a Global Antimicrobial Resistance and Use Surveillance System (GLASS) report, some sources have reported greater than 50% resistance against carbapenem in *K. pneumoniae* isolates (Antimicrobial resistance: global report on surveillance). In our study, there were 214 (27.23%) *K. pneumoniae* isolates found to be resistant to carbapenem. Additionally, our results showed that the susceptibility rates for carbapenems (*P* = 0.0155), cefoperazone-sulbactam (*P* = 0.0003), and amikacin (*P* = 0.0053) were higher for immunocompromised isolates compared to immunocompetent isolates (**Table 2**). Besides, only seven (0.89%) polymyxin resistant *K. pneumoniae* isolates were identified, which is less than findings in studies by others (Podschun and Ullmann., 1998; CDC, 2013; Magill et al., 2014; Martin and Bachman, 2018; Asri et al., 2021). Moreover, MDR-*K. pneumoniae* was more frequently isolated from immunocompromised patients (16.75%) than from immunocompetent patients (10.62%) (*P* = 0.0126).

**Table 2:** Microbiological characteristics of the two study groups (immunocompetent vs immunocompromised).

Characteristic	No./Total (%)		*P* Value
	Immunocompetent patients (n = 386)	Immunocompromised patients (n = 400)	
*Types of sputum culture-positive bacteria*			
One^∀^	247 (63.99%)	193 (48.25%)	<0.0001
Two	107 (27.72%)	146 (36.50%)	0.0084
Three	30 (7.77%)	48 (12.00%)	0.0475
Four	2 (0.52%)	10 (2.50%)	0.0375
Five	0 (0%)	3 (0.75%)	0.2493
*Antimicrobial resistance*			
Carbapenems	90 (23.32%)	124 (31%)	0.0155
Cefoperazone-sulbactam	62 (16.06%)	107 (26.75%)	0.0003
Amikacin	44 (11.40%)	74 (18.50%)	0.0053
Tigecycline	24 (6.22%)	38 (9.50%)	0.0879
Polymyxin	4 (1.04%)	3 (0.75%)	0.7209

^∀^, K. pneumoniae infection.

Data represent No. (%) unless otherwise specified.

### 3.3 Treatment and outcomes

The immunocompromised population has higher rates of antimicrobial resistance and polymicrobial infections. Consequently, there were some differences in case management between the two groups in this study (**Table 3**). The rate of ICU admission (*P* = 0.0125), LOS (*P* < 0.0001), 30-day CFR (*P* < 0.0001), and 6-month CFR (*P* < 0.0001) were all significantly higher in the immunocompromised group (**Table 3**). After propensity score matching, the immunocompromised patients retained a higher risk of death and boasted longer hospital stays (**Supplemental Tables S1**, **S2**).

**Table 3:** Treatment and outcomes of the two study groups (immunocompetent vs immunocompromised).

Treatment	No./Total (%)		*P* Value
	Immunocompetent patients (n = 386)	Immunocompromised patients (n = 400)	
*Treatment*			
Carbapenems	163 (42.23%)	224 (56%)	0.0001
Polymyxin	11 (2.85%)	33 (8.25%)	0.0010
Tigecycline	19 (4.92%)	41 (10.25%)	0.0049
Amikacin	3 (0.78%)	15 (3.75%)	0.0071
Cefoperazone-sulbactam	17 (4.40%)	53 (13.25%)	<0.0001
Sulperazone	44 (11.40%)	85 (21.25%)	0.0002
*Outcomes*			
ICU admission	45 (11.66%)	72 (18.00%)	0.0125
LOS, d, median (IQR)	21 (13-32.25)	28 (16-60)	<0.0001
30-day CFR	25 (6.48%)	97 (24.25%)	<0.0001
6-month CFR	34 (8.81%)	121 (30.25%)	<0.0001

CFR, case fatality rate; ICU, intensive care unit; LOS, length of stay; IQR, interquartile range.

Data represent no. (%) unless otherwise specified.

As is shown in **Table 4**, the absolute neutrophil count (measured on the day or ± one day of sputum culture positive for *K. pneumoniae*) (*P* = 0.0012) and the γGT level (measured on the day of admission) (*P* = 0.0028) were higher in immunocompromised patients than in immunocompetent patients. The time of improvement in immunocompromised patients was 19.21 days, which was later than that in immunocompetent patients (14.70 days, *P* = 0.0010) (**Table 4**). Simultaneously, the levels of creatinine, alanine aminotransferase, aspartate aminotransferase, and γGT changed with statistical significance in both the immunocompetent and immunocompromised groups during treatment (**Table 5**), suggesting the importance the regularly monitoring the liver and renal functions of patients.

**Table 4:** Laboratory values of the two study groups (immunocompetent vs immunocompromised).

Laboratory values	mean ± SEM		*P* Value
	Immunocompetent patients (n = 386)	Immunocompromised patients (n = 400)	
The day or ± one day of sputum culture positive			
PCT, ng/mL	4.345 ± 1.396	4.834 ± 1.085	0.7806
CRP, mg/L	68.32 ± 5.901	57.07 ± 4.116	0.1076
Neu, ×10^9/L	7.205 ± 0.2673	8.721 ± 0.3684	0.0012
Sputum culture turned negative or decreased by two “+”			
times for improvement, d	14.70 ± 0.8380	19.21 ± 1.058	0.0010
PCT, ng/mL	1.086 ± 0.4358	5.152 ± 1.517	0.0293
CRP, mg/L	38.06 ± 4.629	50.73 ± 5.111	0.0730
The day of admission			
Cr, μmol/L	93.16 ± 3.834	103.5 ± 5.724	0.1406
ALT, IU/L	35.89 ± 3.533	46.18 ± 6.099	0.1550
AST, IU/L	46.35 ± 5.706	63.69 ± 10.97	0.1755
γGT, IU/L	55.88 ± 4.847	86.57 ± 8.676	0.0028
The highest value during hospitalization			
Cr, μmol/L	137.5 ± 7.343	165.9 ± 10.29	0.0269
ALT, IU/L	130.2 ± 21.03	172.3 ± 21.68	0.1644
AST, IU/L	204.7 ± 40.07	331.3 ± 54.45	0.0657
γGT, IU/L	106.1 ± 7.323	162.4 ± 12.25	0.0001

PCT, procalcitonin; CRP, C-reactive protein; Neu, neutrophil count; Cr, creatinine; ALT, alanine aminotransferase; AST, aspartate aminotransferase; γGT, gamma-glutamyl transpeptidase.

Data of continuous variables are presented as mean ± SEM (standard error of the mean).

**Table 5:** Changes of laboratory values of the two study groups (immunocompetent vs immunocompromised) during treatment.

Laboratory values	mean ± SEM		*P* Value
	The day of admission	The highest value during treatment	
Immunocompetent patients			
Cr, μmol/L	93.16 ± 3.834	137.5 ± 7.343	<0.0001
ALT, IU/L	35.89 ± 3.533	130.2 ± 21.03	<0.0001
AST, IU/L	46.35 ± 5.706	204.7 ± 40.07	<0.0001
γGT, IU/L	55.88 ± 4.847	106.1 ± 7.323	<0.0001
Immunocompromised patients			
Cr, μmol/L	103.5 ± 5.724	165.9 ± 10.29	<0.0001
ALT, IU/L	46.18 ± 6.099	172.3 ± 21.68	<0.0001
AST, IU/L	63.69 ± 10.97	331.3 ± 54.45	<0.0001
γGT, IU/L	86.57 ± 8.676	162.4 ± 12.25	<0.0001

Cr, creatinine; ALT, alanine aminotransferase; AST, aspartate aminotransferase; γGT, gamma-glutamyl transpeptidase.

Data of continuous variables are presented as mean ± SEM (standard error of the mean).

### 3.4 Logistic regression analysis

In the immunocompromised population, the univariate analysis indicated that age, polymicrobial infection, carbapenem resistance, combined diseases (nervous system diseases, respiratory diseases, cardiovascular diseases, chronic kidney diseases), bacteremia, immunosuppressive agent use, mechanical ventilation, ICU admission, shock, and MDR-*K. pneumoniae infection* corelated with a higher 30-day mortality rate. In the multivariate analysis, respiratory diseases, cardiovascular diseases, mechanical ventilation, and infection with MDR-*K. pneumoniae* were positive predictors of 30-day mortality in the immunocompromised group (**Table 6**). Considering the immunocompetent population, univariate analysis showed that age, polymicrobial infection, carbapenem resistance, combined diseases (nervous system diseases, respiratory diseases, cardiovascular diseases, chronic kidney diseases), mechanical ventilation, and ICU admission represented risk factors for 30-day mortality (**Supplemental Table S3**). However, only mechanical ventilation of immunocompetent patients has been recognized to increase 30-day mortality (**Supplemental Table S3**).

**Table 6:** Logistic regression analysis for variables associated with 30-day mortality of immunocompromised patients infected with *K. pneumoniae*.

variables	univariate		multivariate	
	p value	OR (95% CI)	p value	OR (95% CI)
Age	0.000	1.036 (1.019-1.054)		
Polymicrobial infection	0.000	2.446 (1.509-3.964)		
Resistance to carbapenems	0.000	3.010 (1.864-4.860)		
Nervous system diseases	0.006	2.156 (1.242-3.741)		
Respiratory diseases	0.000	4.655 (2.686-8.066)	0.025	2.189 (1.103-4.344)
Cardiovascular diseases	0.000	3.195 (1.987-5.138)	0.034	2.008 (1.055-3.822)
Chronic kidney diseases	0.000	3.281 (1.810-5.945)		
Bacteremia	0.003	2.551 (1.363-4.775)		
Immunosuppressive agents*	0.001	1.955 (1.296-2.949)		
Mechanical ventilation	0.000	7.491 (4.495-12.486)	0.000	3.982 (2.053-7.722)
ICU admission	0.000	4.058 (2.369-6.954)		
Shock	0.000	7.791 (3.947-15.378)		
MDR-*K. pneumonia*	0.003	2.340 (1.341-4.084)	0.003	3.870 (1.577-9.498)

CI, confidence interval; OR, odds ratio; ICU, intensive care unit; MDR-K. pneumoniae, multi-drug resistant K. pneumoniae.

*, immunosuppressive agents include corticosteroid therapy, biologic modulators, disease-modifying anti-rheumatic drugs or other immunosuppressive drugs.

Separately, univariate analysis indicated that age, polymicrobial infection, carbapenem resistance, combined diseases (nervous system diseases, respiratory diseases, cardiovascular diseases, chronic kidney diseases, chronic hepatic diseases), bacteremia, immunosuppressive agent use, mechanical ventilation, ICU admission, shock, and MDR-*K. pneumoniae infection* were associated with a higher 6-month mortality rate in the immunocompromised group (**Supplemental Table S4**). Separately, the multivariate analysis revealed that respiratory diseases, cardiovascular diseases, immunosuppressive agents, mechanical ventilation, and shock were positive predictors of 6-month mortality in the immunocompromised group (**Supplemental Table S4**). Polymicrobial infection, carbapenem resistance, combined diseases (nervous system diseases, respiratory diseases, cardiovascular diseases), immunosuppressive agent use, blood glucose level, mechanical ventilation, and ICU admission may trigger a higher 6-month mortality rate for immunocompetent patients in the univariate analysis (**Supplemental Table S5**). Finally, nervous system diseases, mechanical ventilation, and ICU admission were predictors of increased 6-month mortality among immunocompetent patients in the multivariate analysis (**Supplemental Table S3**). Predictors of ICU admission for both immunocompetent and immunocompromised patients are listed in **Supplemental Table S6** and **S7**.

## 4 Discussion

The main findings of the present study are as follows. A substantial number of patients hospitalized for *K. pneumoniae* infection in our study were immunocompromised due to various conditions, the most common of which were active malignancy and chronic steroid use. Immunocompromised patients are more likely to develop bacteremia and shock and to require mechanical ventilation support during hospitalization. Compared to immunocompetent patients, immunocompromised patients had a higher probability of polymicrobial infection and a higher rate of antibacterial resistance, which resulted in a worse prognosis with higher ICU admission and mortality rates. Patients in the immunocompromised group with *K. pneumoniae* respiratory infections took more time to improve compared to those in the immunocompetent group. Multivariate analysis indicated that respiratory diseases, cardiovascular diseases, using mechanical ventilation, and being infected with MDR-*K. pneumoniae* led immunocompromised patients to have a higher rate of 30-day mortality. Finally, it is necessary to monitor the liver and renal functions of patients during treatment.

A rapid increase in the drug resistance of *K. pneumoniae* has been witnessed over the past few decades, causing deleterious consequences for infected patients (Martin and Bachman, 2018). It was reported that *K. pneumoniae* has become the most common carbapenem-resistant *Enterobacteriaceae* worldwide because of the use of carbapenem for extended-spectrum β-lactamases infection (Martin and Bachman, 2018). In 2013, the U. S. Center for Disease Control declared carbapenem-resistant *Enterobacteriaceae* to be an urgent threat to public health in the United States, with *K. pneumoniae* present in about 80% of cases (CDC, 2013; Martin and Bachman, 2018). In our study, 27.23% (214/786) of the *K. pneumoniae* isolates were resistant to carbapenems, and 13.74% (108/786) of them were MDR-*K. pneumoniae* isolates, putting our finding in the mid-range level among studies of the same type (Antimicrobial resistance: global report on surveillance; Zhang et al., 2016; Lee et al., 2017; Asri et al., 2021; World Health Organization, 2021; Chen et al., 2022). Since we only enrolled patients with definitive sputum culture results, data on the resistance of *K. pneumoniae* may be slightly biased. However, we still believe this result is of great importance.

Accumulating evidence has demonstrated that resistance to *K. pneumoniae* has reached alarming levels in some areas of the world which indicates that many of the available treatment options for infections in some settings are becoming ineffective. Our results demonstrated that *K. pneumoniae* resistance in the immunocompromised group was significantly more serious than that in the immunocompetent group, which means that we need to perform screening cultures for high-risk patients to guide proper antibiotic use and prevent the misuse of ineffective antibiotics. Polymyxin is a type of antibiotic used to treat Gram-negative infections in the 1960s and 1970s (Jerke et al., 2016). Since then, the renal-toxicity and neurotoxicity of polymyxin have limited its clinical use; however, the emergence of greater *K. pneumoniae* resistance has led clinicians to turn to polymyxin instead as a drug of last resort (Jerke et al., 2016; Liu et al., 2016). Even so, polymyxin is not recommended as an empirical treatment option for *K. pneumoniae* infection unless the antibacterial susceptibility test demonstrates the bacteria are insensitive or resistant to other drugs, such as carbapenems and cefoperazone-sulbactam.

*K. pneumoniae* has been identified as the second most common of bloodstream infections (BSIs) caused by Gram-negative bacteria (Podschun and Ullmann., 1998; Magill et al., 2014). The mortality rate is significantly increased in immunocompromised patients with BSIs involving *K. pneumoniae* (Lin et al., 2022). The reported prevalence of bacteremia ranges from 6%-69%, depending on the pathogens and grade of immunocompromise (Bock et al., 2013; Opota et al., 2015; Taramasso et al., 2016; Ruiz-Giardin et al., 2019). In this study, we found that 48 (12%) immunocompromised patients developed bacteremia, which was associated with ICU admission, 30-day mortality, and 6-month mortality in a univariate analysis (Di Pasquale et al., 2019). Several possible reasons can account for the occurrence of bacteremia in immunosuppressed patients with *K. pneumoniae* infection. The use of invasive devices and procedures, such as mechanical ventilators and catheterization, and exposure to the hospital environment particularly the ICU are common BSIs risk factors (Montrucchio et al., 2022). Respiratory tract, urinary tract, and gastrointestinal colonization are also strongly associated with the risk of BSI. Additionally, various immunocompromised conditions increase the possibility of bacteremia by inhibiting bone marrow proliferation, reducing the activity of immune cells, and damaging the immune barrier. It was reported that the presence of corticosteroid catabolism enzymes in *K. pneumoniae* enhances the ability to use corticosteroids for their own nutrition source, suggesting that the intrinsic bioactivity of *K. pneumoniae* may increase the risk of infection (Chattopadhyay and Banerjee, 2019). Interventions to mitigate BSIs should target all these factors. It is of significance to perform larger-scale analyses or to design more rigorous trials to clarify the route, risk factors, and causative organisms of bacteremia in immunocompromised populations.

In agreement with previous studies, our results indicated that shock occurred more frequently in the immunocompromised group, which was also a risk factor for ICU admission, 30-day CFR, and 6-month CRF in prior univariate analyses (Cillóniz et al., 2011; Sousa et al., 2013; Zhang et al., 2016; Chen et al., 2022). Multivariate analysis has also demonstrated that shock represents a risk factor for ICU admission, 30-day CFR, and 6-month CRF. Thus, shock prevention as well as the timely recognition and treating shock are important to improve the prognosis. Mechanical ventilation is also a key predictor of ICU admission and CFR in both immunocompetent and immunocompromised groups since it implies that the patient developed respiratory failure or other conditions requiring respiratory system support.

Active malignancy and chronic steroid use were the leading immunocompromised factors in our results, which is consistent with findings of previous studies (Di Pasquale et al., 2019; Ramirez et al., 2020; Certan et al., 2022). Additionally, we suggest that patients may have more than one risk factor characteristic. Thus, clinical assessment should be comprehensive, taking into consideration risk factors for immunosuppression and their associated biological mechanisms.

Numerous studies have shown that the *Streptococcus pneumoniae* vaccine and influenza virus vaccine play an important role in clinical practice. Likewise, we believe that a *K. pneumoniae* vaccine is a feasible and promising strategy for *K. pneumoniae* infection. However, due to the complex drug resistance mechanism of *K. pneumoniae*, the development of its vaccine has been slow (Assoni et al., 2021). Although various vaccine candidates against *K. pneumoniae* have been proposed, there are no vaccines available on the market currently. Therefore, as far as the current situation is concerned, early diagnosis and proper treatment of *K. pneumoniae* infection are of great importance.

The current study has some limitations. First, we were not able to involve many investigators from more clinical centers, thus limiting the generalizability of our findings. However, our findings are valuable given the high prevalence of *K. pneumoniae* globally and the increase in the immunocompromised population. Another major limitation is the infeasibility of grading the severity of immunocompromised patients and, therefore, stratifying patients and defining the physio-pathological interactions between different risk factors, especially regarding the use of biological drugs and chronic steroids. Additionally, our study did not involve infections caused by fungi, viruses, and other pathogens. Previous studies have indicated that immunocompromised populations are vulnerable to various pathogens (Sousa et al., 2013; Di Pasquale et al., 2019; Certan et al., 2022). The prognosis of infectious diseases is the result of many factors related to the patient, pathogen, and treatment. The existence of other infectious pathogens, especially severe acute respiratory syndrome coronavirus 2, may have been an important confounding factor in our study that may have contributed to the mortality rate. This was a retrospective study and recent, rapidly evolving scenarios might have changed the situation, especially considering the coronavirus disease 2019 pandemic and the introduction of new antibiotics for multi-resistant pathogens. Future multicenter and prospective studies on patients with specific characteristics of immunosuppression could provide practical recommendations to confirm our data and better define the impact of each risk factor individually.

In conclusion, compared to the immunocompetent population, immunocompromised patients were more likely to develop bacteremia and shock and showed a greater probability of polymicrobial infection and a higher rate of resistance, resulting in worse prognosis with higher ICU admission and mortality rates. Multivariate analysis indicated that respiratory diseases, cardiovascular diseases, using mechanical ventilation, and infection with MDR-*K. pneumoniae* were more likely to cause a higher rate of 30-day mortality in the immunocompromised population. Based on this study, we conclude that rational use of anti-infective drugs, reduction of invasive procedures, strict implementation of aseptic techniques, and comprehensive intervention for comorbidities for hospitalized patients can significantly improve the outcomes.

## Data availability statement

The original contributions presented in the study are included in the article/**Supplementary Material**. Further inquiries can be directed to the corresponding authors.

## Ethics statement

The studies involving human participants were reviewed and approved by Ruijin Hospital Affiliated to Shanghai Jiaotong University School of Medicine Ethics Committee. Written informed consent to participate in this study was provided by the participants’ legal guardian/next of kin.

## Author contributions

Conception and design of the work: YF, YX, and GS. Data interpretation: YF and GS. Collecting data: YL, LH, JC, HZ, JL, and YY. Data analysis: YL, LH, and JC. Drafting the work and revising it critically for important intellectual content: YL, LH, JC, GS, and YF. All authors contributed to the article and approved the submitted version.

## References

[B1] Asri N. A. M. Ahmad S. Mohamud R. Hanafi M. Zaidi N. F. M. Irekeola A. A.. (2021). Global prevalence of nosocomial multidrug-resistant Klebsiella pneumoniae: a systematic review and meta-analysis. Antibiotics. 10 (12), 1508. doi: 10.3390/antibiotics10121508 34943720 PMC8698758

[B2] Assoni L. Girardello R. Converso T. R. Darrieux M. (2021). Current stage in the development of Klebsiella pneumoniae vaccines. Infect. Dis. Ther. 10 (4), 2157–2175. doi: 10.1007/s40121-021-00533-4 34476772 PMC8412853

[B3] Bock A. M. Cao Q. Ferrieri P. Young J. A. H. Weisdorf D. J. (2013). Bacteremia in blood or marrow transplantation patients: clinical risk factors for infection and emerging antibiotic resistance. Biol. Blood Marrow Transplant. 19 (1), 102–108. doi: 10.1016/j.bbmt.2012.08.016 22940054

[B4] Centers for Disease Control and Prevention (U.S.). (2013). Antibiotic resistance threats in the United States (Atlanta, GA: U.S. Department of Health and Human Services, Centers for Disease Control and Prevention). doi: 10.1016/j.medmal.2007.05.006

[B5] Certan M. Garcia Garrido H. M. Wong G. Heijmans J. Grobusch M. P. Goorhuis A. (2022). Incidence and predictors of community-acquired pneumonia in patients with hematological cancers between 2016 and 2019. Clin. Infect. Dis. 75 (6), 1046–1053. doi: 10.1093/cid/ciac005 35195716 PMC9522390

[B6] Chattopadhyay P. Banerjee G. (2019). Corticosteroid catabolism by Klebsiella pneumoniae as a possible mechanism for increased pneumonia risk. Curr. Pharm. Biotechnol. 20 (4), 309–316. doi: 10.2174/1389201020666190313153841 30868949

[B7] Chen I-R. Lin S-N. Wu X-N. Chou S-H. Wang F-D. Lin Y-T. (2022). Clinical and microbiological characteristics of bacteremic pneumonia caused by Klebsiella pneumoniae. Front. Cell Infect. Microbiol. 12, 903682. doi: 10.3389/fcimb.2022.903682 35811668 PMC9259976

[B8] Cillóniz C. Ewig S. Ferrer M. Polverino E. Gabarrús A. Puig de la Bellacasa J.. (2011). Community-acquired polymicrobial pneumonia in the intensive care unit: aetiology and prognosis. Crit. Care 15 (5), R209. doi: 10.1186/cc10444 21914220 PMC3334753

[B9] CLSI. (2021). Performance standards for antimicrobial susceptibility testing, M100. 31st ed (Wayne, PA: Clinical and Laboratory Standards Institute). 10.1128/JCM.00213-21 PMC8601225 34550809

[B10] Di Pasquale M. F. Sotgiu G. Gramegna A. Radovanovic D. Terraneo S. Reyes L. F.. (2019). Prevalence and etiology of community-acquired pneumonia in immunocompromised patients. Clin. Infect. Dis. 68 (9), 1482–1493. doi: 10.1093/cid/ciy723 31222287 PMC6481991

[B11] Jerke K. H. Lee M. J. Humphries R. M. (2016). Polymyxin susceptibility testing: a cold case reopened. Clin. Microbiol. Newsl. 38 (9), 69–77. doi: 10.1016/j.clinmicnews.2016.04.003

[B12] Juan C-H. Chuang C. Chen C-H. Li L. Lin. Y-T. (2019). Clinical characteristics, antimicrobial resistance and capsular types of community-acquired, healthcare-associated, and nosocomial Klebsiella pneumoniae bacteremia. Antimicrob. Resist. Infect. Control. 8 (1), 1–9. doi: 10.1186/s13756-018-0426-x 30622702 PMC6318907

[B13] Lee C-R. Lee J. H. Park K. S. Jeon J. H. Kim Y. B. Cha C-J.. (2017). Antimicrobial resistance of hypervirulent Klebsiella pneumoniae: epidemiology, hypervirulence-associated determinants, and resistance mechanisms. Front. Cell Infect. Microbiol. 7, 483. doi: 10.3389/fcimb.2017.00483 29209595 PMC5702448

[B14] Lin Y. T. Jeng Y. Y. Chen T. L. Fung C. P. (2010). Bacteremic community-acquired pneumonia due to Klebsiella pneumoniae: clinical and microbiological characteristics in Taiwan, 2001-2008. BMC Infect. Dis. 10 (1), 307. doi: 10.1186/1471-2334-10-307 20973971 PMC2987304

[B15] Lin H. X. Yang L. L. Fang J. Gao Y. L. Zhu H. X. Zhang S. X.. (2022). Clinical characteristics of bloodstream infection in immunosuppressed patients: a 5-year retrospective cohort study. Front. Cell Infect. Microbiol. 12, 796656. doi: 10.3389/fcimb.2022.796656 35444962 PMC9014008

[B16] Liu Y. Y. Wang Y. Walsh T. R. Yi L. X. Zhang R. Spencer J.. (2016). Emergence of plasmid-mediated colistin resistance mechanism MCR-1 in animals and human beings in China: a microbiological and molecular biological study. Lancet Infect. Dis. 16 (2), 161–168. doi: 10.1016/S1473-3099(15)00424-7 26603172

[B17] Magill S. S. Edwards J. R. Bamberg W. Beldavs Z. G. Dumyati G. Kainer M. A.. (2014). Multistate point-prevalence survey of health care–associated infections. N Engl. J. Med. 370 (13), 1198–1208. doi: 10.1056/nejmoa1306801 24670166 PMC4648343

[B18] Martin R. M. Bachman M. A. (2018). Colonization, infection, and the accessory genome of Klebsiella pneumoniae. Front. Cell Infect. Microbiol. 8, 4. doi: 10.3389/fcimb.2018.00004 29404282 PMC5786545

[B19] Melot B. Colot J. Guerrier G. (2015). Bacteremic community-acquired infections due to Klebsiella pneumoniae: clinical and microbiological presentation in New Caledonia, 2008-2013. Int. J. Infect. Dis. 41, 29–31. doi: 10.1016/j.ijid.2015.10.013 26518064

[B20] Montrucchio G. Costamagna A. Pierani T. Petitti A. Sales G. Pivetta E.. (2022). Bloodstream infections caused by carbapenem-resistant pathogens in intensive care units: risk factors analysis and proposal of a prognostic score. Pathogens. 11 (7), 718. doi: 10.3390/pathogens11070718 35889963 PMC9315650

[B21] Opota O. Croxatto A. Prod’hom G. Greub G. (2015). Blood culture-based diagnosis of bacteraemia: State of the art. Clin. Microbiol. Infect. 21 (4), 313–322. doi: 10.1016/j.cmi.2015.01.003 25753137

[B22] Podschun R. Ullmann U. (1998). Klebsiella spp. as nosocomial pathogens: epidemiology, taxonomy, typing methods, and pathogenicity factors. Clin. Microbiol. Rev. 11, 589–603. doi: 10.1128/CMR.11.4.589 9767057 PMC88898

[B23] Ramirez J. A. Musher D. M. Evans S. E. Dela Cruz C. Crothers K. A. Hage C. A.. (2020). Treatment of community-acquired pneumonia in immunocompromised adults: a consensus statement regarding initial strategies. Chest. 158 (5), 1896–1911. doi: 10.1016/j.chest.2020.05.598 32561442 PMC7297164

[B24] Ruiz-Giardin J. M. Ochoa Chamorro I. Velázquez Riós L. Jaqueti Aroca J. García Arata M. I. SanMartín López J. V.. (2019). Blood stream infections associated with central and peripheral venous catheters. BMC Infect. Dis. 19 (1), 841. doi: 10.1186/s12879-019-4505-2 31615450 PMC6794764

[B25] Shankar C. Veeraraghavan B. Nabarro L. E. B. Ravi R. Ragupathi N. K. D. Rupali P. (2018). Whole genome analysis of hypervirulent Klebsiella pneumoniae isolates from community and hospital acquired bloodstream infection. BMC Microbiol. 18 (1), 6. doi: 10.1186/s12866-017-1148-6 29433440 PMC5809863

[B26] Sousa D. Justo I. Domínguez A. Manzur A. Izquierdo C. Ruiz L.. (2013). Community-acquired pneumonia in immunocompromised older patients: incidence, causative organisms and outcome. Clin. Microbiol. Infect. 19 (2), 187–192. doi: 10.1111/j.1469-0691.2012.03765.x 22390624

[B27] Taramasso L. Tatarelli P. Di Biagio A. (2016). Bloodstream infections in HIV-infected patients. Virulence. 7 (3), 320–328. doi: 10.1080/21505594.2016.1158359 26950194 PMC4871667

[B28] World Health Organization (2021) Global antimicrobial resistance and use surveillance system (GLASS) report 2021. Available at: http://www.who.int/glass/resources/publications/early-implementation-report-2020/en/.

[B29] World Health Organization. (2014). Antimicrobial resistance: global report on surveillance. Available at: https://www.who.int/publications/i/item/9789241564748.

[B30] Zhang Y. Zhao C. Wang Q. Wang X. Chen H. Li H.. (2016). High prevalence of hypervirulent Klebsiella pneumoniae infection in China: geographic distribution, clinical characteristics, and antimicrobial resistance. Antimicrob. Agents Chemother. 60 (10), 6115–6120. doi: 10.1128/AAC.01127-16 27480857 PMC5038323

