# Supplementary material for: Clinical characteristics of respiratory tract infection caused by Klebsiella pneumoniae in immunocompromised patients: a retrospective cohort study
Source: Front Cell Infect Microbiol. 2023 Aug 16;13:1137664. doi: 10.3389/fcimb.2023.1137664 (PMC10469001; doi:10.3389/fcimb.2023.1137664)
Supplement: Supplementary file 1 [file DataSheet_1.docx]

Supplementary Material

Clinical Characteristics of Respiratory Tract Infection Caused by *Klebsiella pneumoniae* in Immunocompromised Patients: A Retrospective Cohort Study

**Yahui Liu^1,2†^, Lin Huang^1,2†^, Jing Cai^3†^, Haixing Zhu^1,2^, Junjie Li^1,2^, Youchao Yu^1,2^, Yumin Xu^4*^, Guochao Shi^1,2*^, Yun Feng^1,2*^**

^1^Department of Respiratory and Critical Care Medicine, Ruijin Hospital Affiliated Shanghai Jiao Tong University School of Medicine, Shanghai, China, ^2^Institute of Respiratory Diseases, School of Medicine, Shanghai Jiaotong University, Shanghai, China,^3^Department of Pulmonary and Critical Care Medicine, Haining People's Hospital, Jiaxing, China, ^4^Department of Hospital Infection Management, Department of Infectious Diseases, Ruijin Hospital, School of Medicine, Shanghai Jiao Tong University, Shanghai, China

*** Correspondence:**Corresponding Author

Yumin Xu: E-mail, xym121@163.com;

Guochao Shi: E-mail, shiguochao@hotmail.com;

Yun Feng: E-mail, fy01057@163.com;

^†^These authors have contributed equally to this work and share first authorship.

# Supplementary Figures and Tables

## Supplementary Figures

**
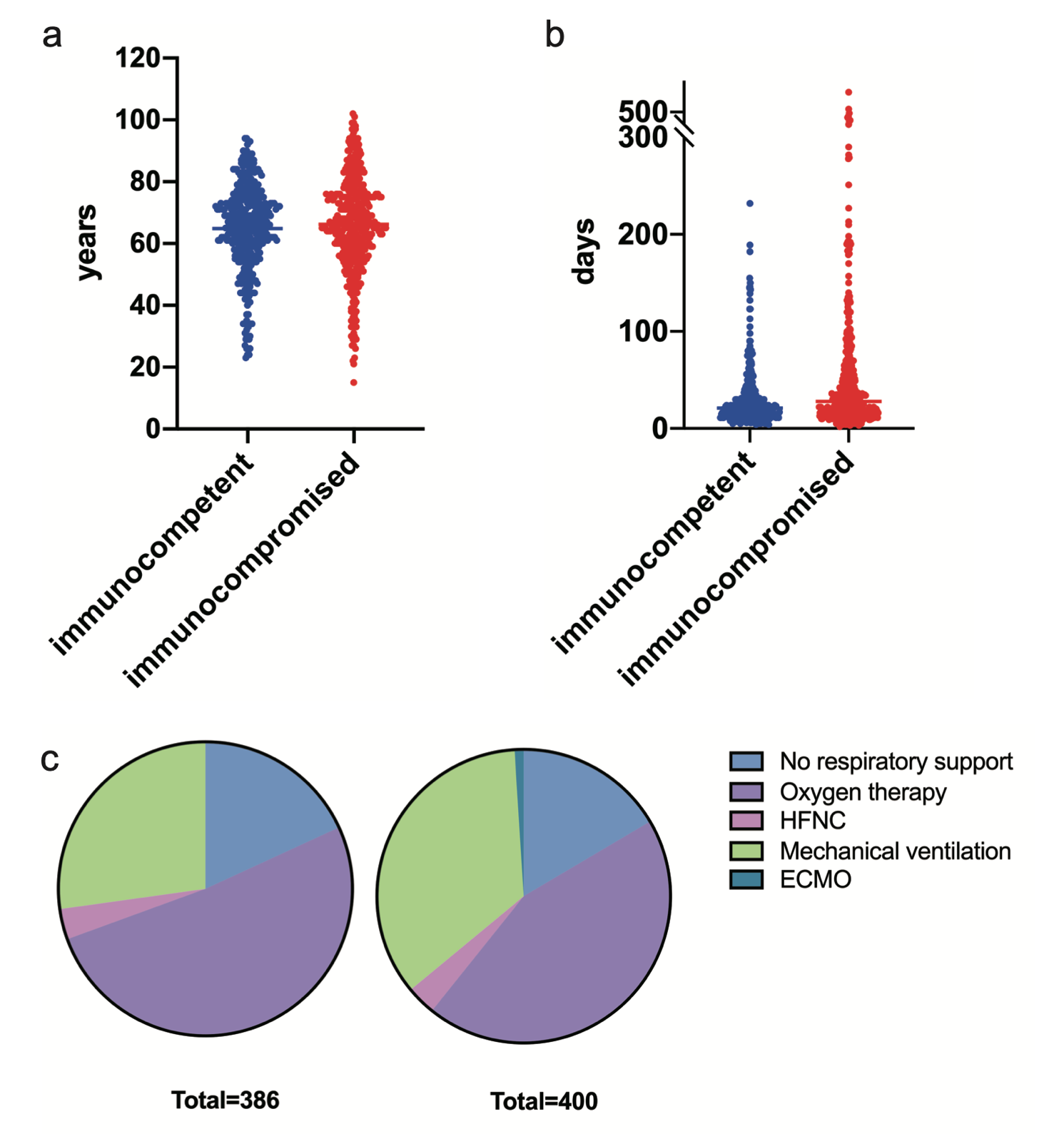
**

**Supplementary Figure 1.** (a) The distributions of age of the immunocompetent and immunocompromised patients. (b) The distribution of length of stay (LOS) of the immunocompetent and immunocompromised patients. (c) Respiratory support of the immunocompetent (left) and immunocompromised (right) patients.

**
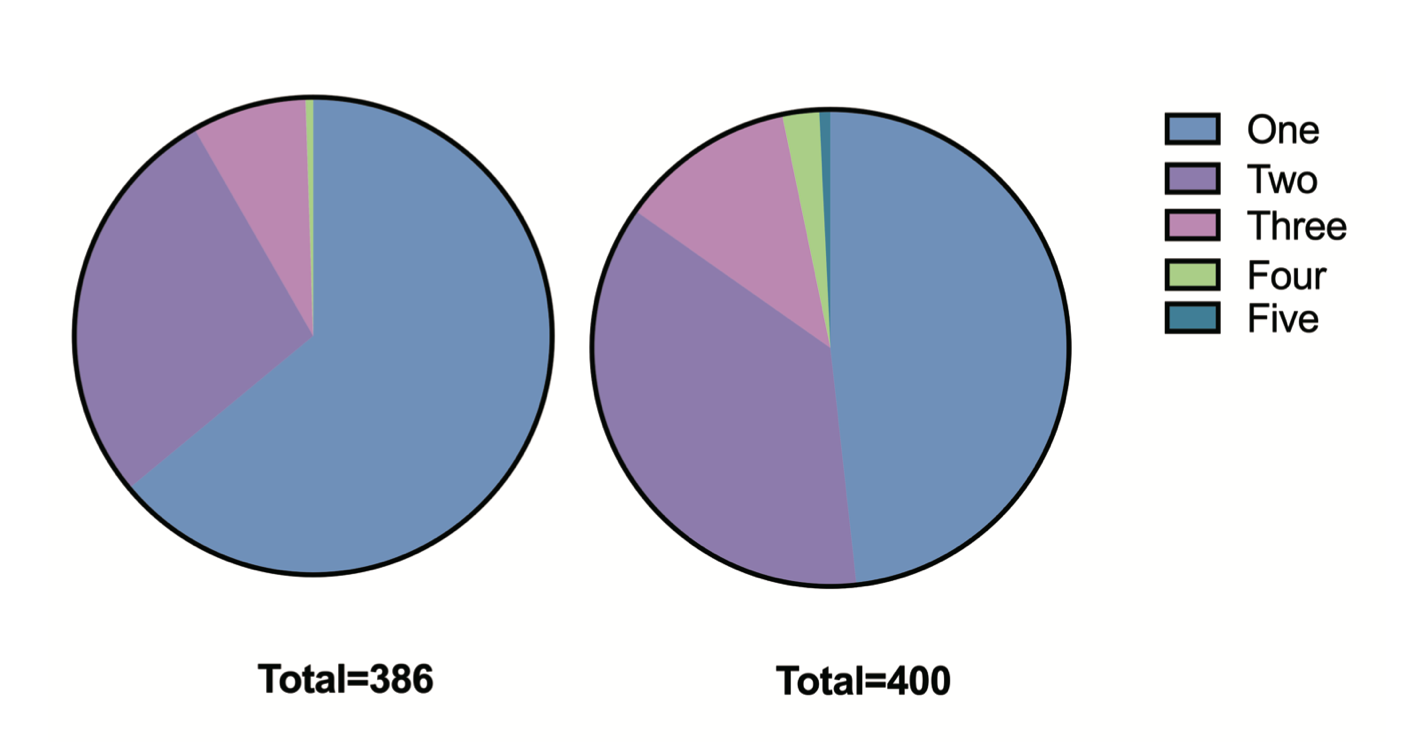
**

**Supplementary Figure 2.** The distribution of types of sputum culture-positive bacteria in immunocompetent (left) and immunocompromised (right) patients.

## Supplementary Tables

Table S1. Baseline characteristic of the immunocompetent and immunocompromised patients

| Characteristic | | Before PSM (No./Total (%)) | | *P* Value | After PSM (No./Total (%)) | | *P* Value |
| --- | --- | --- | --- | --- | --- | --- | --- |
|  |  | Immunocompetent patients (n=386) | Immunocompromised patients (n=400) |  | Immunocompetent patients (n=232) | Immunocompromised patients (n=232) |  |
| *Mechanical ventilation* |  | 105 (27.20%) | 140 (35.00%) | 0.0183 | 62 (26.72%) | 64 (27.58%) | 0.835 |
| *Invasive operation or surgery* |  | 259 (67.10%) | 163 (40.75%) | <0.0001 | 126 (54.31%) | 129 (55.60%) | 0.780 |
| *Comorbid conditions* | Respiratory diseases | 173 (44.82%) | 220 (55.00%) | 0.0043 | 107 (46.12%) | 110 (47.41%) | 0.780 |
|  | Diabetes | 73 (18.91%) | 102 (25.50%) | 0.0265 | 38 (16.38%) | 38 (16.38%) | 1 |
|  | History of solid organ tumor | 103 (26.68%) | 193 (48.25%) | <0.0001 | 100 (43.10%) | 101 (43.53%) | 0.925 |
|  | Chronic kidney disease | 21 (5.44%) | 54 (13.50%) | 0.0045 | 14 (6.03%) | 16 (6.90%) | 0.706 |
|  | Chronic hepatic diseases | 10 (2.59%) | 41 (10.25%) | <0.0001 | 10 (4.31%) | 9 (3.88%) | 0.815 |

PSM, propensity score matching.

Data represent no. (%) unless otherwise specified.

Table S2. Outcomes of the immunocompetent and immunocompromised patients

| Outcomes | Before PSM (No./Total (%)) | | *P* Value | After PSM (No./Total (%)) | | *P* Value |
| --- | --- | --- | --- | --- | --- | --- |
|  | Immunocompetent patients (n=386) | Immunocompromised patients (n=400) |  | Immunocompetent patients (n=232) | Immunocompromised patients (n=232) |  |
| ICU admission | 45 (11.66%) | 72 (18.00%) | 0.0125 | 21(9.05%) | 23 (9.91%) | 0.751 |
| LOS, d, median (IQR) | 21 (13-32.25) | 28 (16-60) | <0.0001 | 20.5 (13-33) | 25 (15-51) | 0.001 |
| 30-day CFR | 25 (6.48%) | 97 (24.25%) | <0.0001 | 15 (6.47%) | 23 (9.91%) | 0.009 |
| 6-month CFR | 34 (8.81%) | 121 (30.25%) | <0.0001 | 19 (8.19%) | 41 (17.67%) | 0.002 |

PSM, propensity score matching; ICU, intensive care unit; LOS, length of stay; IQR, interquartile range; CFR, case fatality rate.

Data represent no. (%) unless otherwise specified.

Table S3. Logistic regression analysis for variables associated with 30-day mortality of immunocompetent patients infected with *K. pneumoniae*

| variables | univariate | | multivariate | |
| --- | --- | --- | --- | --- |
|  | p value | OR (95% CI) | p value | OR (95% CI) |
| Age | 0.021 | 1.041 (1.006-1.076) |  |  |
| Polymicrobial infection | 0.090 | 2.021 (0.895-4.560) |  |  |
| Resistance to carbapenems | 0.014 | 2.821 (1.232-6.459) |  |  |
| Nervous system diseases | 0.000 | 4.694 (2.049-10.753) |  |  |
| Respiratory diseases | 0.007 | 3.418 (1.393-8.385) |  |  |
| Cardiovascular diseases | 0.035 | 2.942 (1.080-8.014) |  |  |
| Chronic kidney diseases | 0.024 | 3.854 (1.190-12.480) |  |  |
| Mechanical ventilation | 0.000 | 10.126 (3.919-26.163) | 0.000 | 9.727 (3.091-30.606) |
| ICU admission | 0.054 | 2.607 (0.982-6.920) |  |  |

CI, confidence interval; OR, odds ratio; ICU, intensive care unit.

Table S4. Logistic regression analysis for variables associated with 6-month mortality of immunocompromised patients infected with *K. pneumoniae*

| variables | univariate | | multivariate | |
| --- | --- | --- | --- | --- |
|  | p value | OR (95% CI) | p value | OR (95% CI) |
| Age | 0.000 | 1.045 (1.028-1.064) |  |  |
| Polymicrobial infection | 0.000 | 2.59 (1.654-4.056) |  |  |
| Resistance to carbapenems | 0.000 | 3.556 (2.253-5.612) |  |  |
| Nervous system diseases | 0.005 | 2.12 (1.247-3.602) |  |  |
| Respiratory diseases | 0.000 | 5.483 (3.286-9.15) | 0.015 | 2.355 (1.177-4.709) |
| Cardiovascular diseases | 0.000 | 3.736 (2.387-5.847) | 0.011 | 2.384 (1.221-4.653) |
| Chronic kidney diseases | 0.000 | 4.2 (2.319-7.607) |  |  |
| Chronic hepatic diseases | 0.020 | 2.176 (1.13-4.19) |  |  |
| bacteremia | 0.000 | 3.899 (2.096-7.254) |  |  |
| Immunosuppressive agents* | 0.001 | 1.913 (1.295-2.825) | 0.047 | 4.257 (1.016-17.831) |
| Mechanical ventilation | 0.000 | 10.619 (6.467-7.436) | 0.000 | 6.027 (3.021-12.023) |
| ICU admission | 0.000 | 5.122 (2.99-8.775) |  |  |
| Shock | 0.000 | 13.786 (6.161-30.850) | 0.004 | 4.608 (1.634-12.996) |
| MDR-*K. pneumoniae* | 0.000 | 2.913 (1.702-4.988) |  |  |

CI, confidence interval; OR, odds ratio; ICU, intensive care unit; MDR-*K. pneumoniae*, multi-drug resistant *K. pneumoniae*.

*, immunosuppressive agents include corticosteroid therapy, biologic modulators, disease-modifying anti-rheumatic drugs or other immunosuppressive drugs.

Table S5. Logistic regression analysis for variables associated with 6-month mortality of immunocompetent patients infected with *K. pneumoniae*

| variables | univariate | | multivariate | |
| --- | --- | --- | --- | --- |
|  | p value | OR (95% CI) | p value | OR (95% CI) |
| Polymicrobial infection | 0.005 | 2.797 (1.365-5.734) |  |  |
| Resistance to carbapenems | 0.012 | 2.557 (1.233-5.302) |  |  |
| Nervous system diseases | 0.001 | 3.283 (1.598-6.746) | 0.030 | 2.482 (1.091-5.645) |
| Respiratory diseases | 0.017 | 2.440 (1.171-5.086) |  |  |
| Cardiovascular diseases | 0.015 | 2.898 (1.229-6.832) |  |  |
| Immunosuppressive agents* | 0.044 | 2.119 (1.019-4.408) |  |  |
| Blood glucose | 0.082 | 1.008 (0.989-1.197) |  |  |
| Mechanical ventilation | 0.000 | 11.231 (4.892-25.783) | 0.000 | 5.937 (2.239-15.745) |
| ICU admission | 0.000 | 5.273 (2.394-11.611) | 0.025 | 3.038 (1.147-8.048) |

CI, confidence interval; OR, odds ratio; ICU, intensive care unit.

*, immunosuppressive agents include corticosteroid therapy, biologic modulators, disease-modifying anti-rheumatic drugs or other immunosuppressive drugs.

Table S6. Logistic regression analysis for variables associated with ICU admission of immunocompromised patients infected with *K. pneumoniae*

| variables | univariate | | multivariate | |
| --- | --- | --- | --- | --- |
|  | p value | OR (95% CI) | p value | OR (95% CI) |
| Age | 0.062 | 0.981 (0.960-1.001) |  |  |
| Polymicrobial infection | 0.011 | 2.246 (1.2-4.205) |  |  |
| Resistance to carbapenems | 0.001 | 3.119 (1.637-5.943) |  |  |
| MDR-*K. pneumoniae* | 0.002 | 3.354 (1.543-7.289) |  |  |
| Chronic kidney diseases | 0.018 | 3.344 (1.226-9.119) | 0.039 | 3.018 (1.055-8.630) |

CI, confidence interval; OR, odds ratio; MDR-*K. pneumoniae*, multi-drug resistant *K. pneumoniae*.

Table S7. Logistic regression analysis for variables associated with ICU admission of immunocompetent patients infected with *K. pneumoniae*

| variables | univariate | | multivariate | |
| --- | --- | --- | --- | --- |
|  | p value | OR (95% CI) | p value | OR (95% CI) |
| Age | 0.092 | 1.015 (0.998-1.032) |  |  |
| Polymicrobial infection | 0.003 | 2.292 (1.335-3.936) |  |  |
| Resistance to carbapenems | 0.000 | 3.187 (1.88-5.401) |  |  |
| Nervous system diseases | 0.030 | 1.947 (1.065-3.559) |  |  |
| Respiratory diseases | 0.001 | 2.689 (1.525-4.741) |  |  |
| Cardiovascular diseases | 0.001 | 2.471 (1.467-4.162) | 0.038 | 2.068 (1.043-4.101) |
| Chronic kidney diseases | 0.007 | 2.431 (1.279-4.622) |  |  |
| Bacteremia | 0.000 | 5.111 (2.69-9.71) |  |  |
| Mechanical ventilation | 0.000 | 7.265 (4.079-12.938) | 0.000 | 3.906 (1.907-8) |
| Shock | 0.000 | 9.161 (4.645-18.066) | 0.001 | 4.345 (1.892-9.981) |
| MDR-*K. pneumoniae* | 0.017 | 2.091 (1.14-3.837) |  |  |

CI, confidence interval; OR, odds ratio; ICU, intensive care unit; MDR-*K. pneumoniae*, multi-drug resistant *K. pneumoniae*.
